# Racial disparity and regional variance in healthcare utilization among patients with lung cancer in US hospitals during 2016–2019

**DOI:** 10.1186/s13690-023-01166-4

**Published:** 2023-08-17

**Authors:** Jongwha Chang, Mar Medina, Dong Yeong Shin, Sun Jung Kim

**Affiliations:** 1 Department of Pharmaceutical Sciences, Irma Lerma Rangel School of Pharmacy, Texas A&M University, College Station, TX USA; 2 School of Pharmacy, University of Texas at El Paso, El Paso, TX USA; 3 Department of Public Health Sciences, College of Health and Social Services, New Mexico State University, Las Cruces, NM USA; 4 Department of Health Administration and Management, College of Medical Science, Soonchunhyang University, Asan, Republic of Korea; 5 Center for Healthcare Management Science, Soonchunhyang University, Asan, Republic of Korea; 6 Department of Software Convergence, Soonchunhyang University, Asan, Republic of Korea

**Keywords:** Lung cancer, NIS sample, Healthcare utilization, Racial disparity, Regional variance

## Abstract

**Background:**

Lung cancer health disparities are related to various patient factors. This study describes regional differences in healthcare utilization and racial characteristics to identify high-risk areas. This study aimed to identify regions and races at greater risk for lung cancer health disparities based on differences in healthcare utilization, measured here by hospital charges and length of stay.

**Methods:**

The National Inpatient Sample of the United States was used to identify patients with lung cancer (n = 92,159, weighted n = 460,795) from 2016 to 2019. We examined the characteristics of the patient sample and the association between the racial and regional variables and healthcare utilization, measured by hospital charges and length of stay. The multivariate sample weighted linear regression model estimated how racial and regional variables are associated with healthcare utilization.

**Results:**

Out of 460,795 patients, 76.4% were white, and 40.2% were from the South. The number of lung cancer patients during the study periods was stable. However, hospital charges were somewhat increased, and the length of stay was decreased during the study period. Sample weighted linear regression results showed that Hispanic & Asian patients were associated with 21.1% and 12.3% higher hospital charges than White patients. Compared with the Northeast, Midwest and South were associated with lower hospital charges, however, the West was associated with higher hospital charges.

**Conclusion:**

Minority groups and regions are at an increased risk for health inequalities because of differences in healthcare utilization. Further differences in utilization by insurance type may exacerbate the situation for some patients with lung cancer. Hospital managers and policymakers working with these patient populations in identified areas should strive to address these disparities through special prevention programs and targeted financial assistance.

**Table:** 

**Text box 1. Contributions to the literature**
• Researches has shown that the critical health disparities for lung cancer in terms of incidence, treatment, mortality, however, lack of research presented by healthcare utilization.
• We found evidences of healthcare utilization disparities by race, region, and other socioeconomic status. Results of this study suggest ideas of targeted prevention programs to those vulnerable patient groups.
• These findings contribute to fill the gaps in the current literature and deliver strong message for not only achieving health equity but also further research to identify closing the disparity gap to increase access and the health of disadvantaged communities and at-risk regions.

## Background

There were 1.8 million worldwide lung cancer deaths in 2020 [1]. In 2022, lung cancer was the third most common cancer and the leading cause of cancer death in the United States, with about 236,740 new cases and 130,180 deaths [2]. Lung cancer was the cause of approximately 12.3% of new cancer cases in 2022 and 21.4% of cancer deaths in 2022 [2]. Despite advances in tobacco control, the five-year survival rate for lung cancer (from 2012 to 2018) was 22.9% [2, 3]. Incidence is higher in patients between 65 and 74 years old and African American men[2]. Treatment for lung cancer varies by patient, but surgical options and minor invasive procedures have the best survival rates [4, 5]. However, patients are less likely to undergo surgery if they are low income, have low education, live in rural areas, are uninsured, or have Medicaid[5]. Because of the high prevalence of lung cancer in the United States and worldwide, it is necessary to characterize health disparities by healthcare utilization and location to identify areas and populations with greater needs.

Differences in healthcare utilization that have severe implications for patient outcomes appear in the emergency and inpatient settings. At the end of life, minority patients with lung cancer utilized healthcare more and spent more for more intensive care that did not improve outcomes [6, 7]. African American and Asian patients had higher inpatient costs than white patients, who were likelier to use cheaper outpatient and hospice care[6]. Another study found similar results, with minority patients with cancer more likely to receive care at their end of life from emergency rooms, intensive care units (ICU), and inpatient facilities[7]. ICU utilization, typically costly, was increased in minority patients, with African Americans and Asians often charged more than other racial or ethnic groups[6]. Asian Americans, in one study, were more likely to spend longer in the ICU than Caucasian patients[6].

Lung cancer is also the leading cause of cancer-related emergency room visits, with patients often presenting with pain problems[8]. Such visits are significant because cancer-related emergency room visits often reflect poor cancer care[9]. Those visits also often result in hospitalization or transfer to a skilled nursing facility, intermediate care facility, or home healthcare[8]. For example, African Americans and Hispanics are less likely to go to a skilled nursing facility or intermediate care facility than Caucasian patients[8]. Previous research characterized how Hispanic patients tend to be treated in the ER and ICU with more aggressive chemotherapy use at their end of life, increasing cost[7].

With lung cancer having a nationwide cost of $20.1 billion and expected to rise by 33–36% by 2030, [10, 11] hospital charges become a crucial barrier to care. Socioeconomic status (SES) is affected by income, education, race, insurance, and environment, with low SES related to low surgery rates for non-small cell lung cancer[12]. According to Ebner et al., lack of or nonstandard treatment is connected to low income, low education, Medicaid or no insurance, and rural residence[12]. Similarly, David et al. found that patients with a lack of insurance, inadequate education, and low income often did not receive treatment for later-stage lung cancer[13].

Regional variances in health disparities for patients with lung cancer have also started to emerge and are essential to understand to promote targeted change in these areas. Regarding mortality rates, Mukherjee et al. found that the Northeastern states of the US had the lowest risk of death[4]. In contrast, the West had worse survival rates and higher mortality-to-incidence ratios[4]. These results were echoed by Bick et al., who found more significant treatment disparities in the West South Central, which also had a large minority population[14]. Better outcomes have typically been connected to regions with more insured patients, a white population, and higher socioeconomic status[14]. In that study, the Middle Atlantic had higher insurance and income levels, minor treatment disparities and mortality rates, and excellent survival rates[14].

Differences can also be found between urban and rural areas. Rural areas have higher smoking rates, [15] leading to almost double lung cancer deaths than urban regions [16, 17]. Rural patients have higher smoking rates than urban areas, but there is no difference in overall cigarette exposure between Black and white rural patients[15]. Still, rural black patients have increased non-small cell lung cancer rates[15]. Rural patients also utilize surgery less than urban patients, possibly due to lack of access to specialists or distance from care[16]. There are higher rates of poverty, lack of insurance, low income, low education, and older age in rural areas, and these factors are prevalent in the South, which has the most outstanding rates of smoking and lung cancer [17, 18].

The American Thoracic Society acknowledged critical health disparities for lung cancer regarding “incidence, diagnosis, treatment, and mortality,” with incidence and mortality varying by “race, ethnicity, sex, and SES.” [19] Previous research has highlighted that lung cancer health disparities are related to various patient factors. This study describes racial and regional differences in healthcare utilization to identify high-risk populations. Those populations may benefit from increased resources and surveillance to prevent and help treat lung cancer based on the risk factors found in this study. Healthcare utilization here is defined by hospital charges and length of stay, with higher costs and length of stay between races and geographic regions demonstrating health disparities.

## Methods

### Data collection

**Fig. 1:**
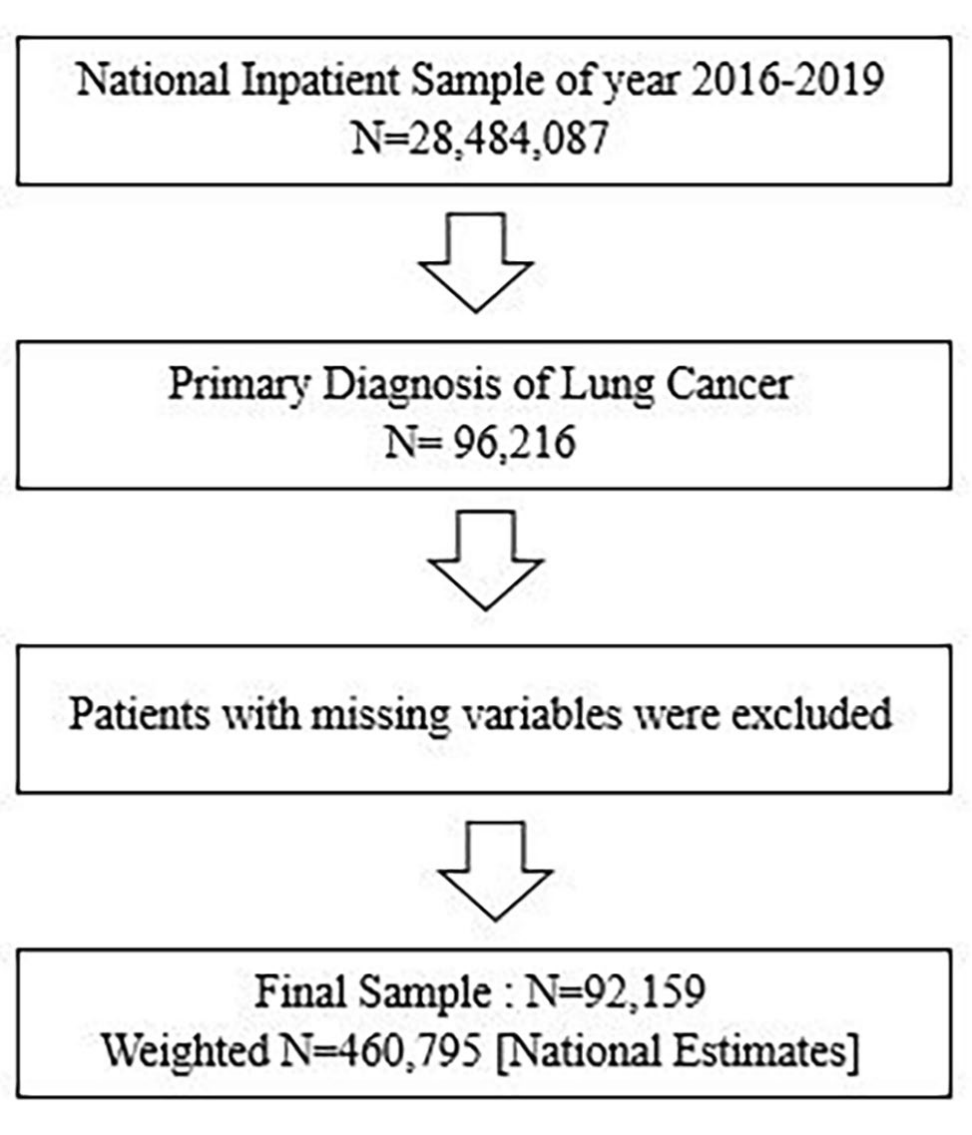
Flow chart of study sample selection

The latest 2016–2019 United States National Inpatient Sample (NIS) data was used to obtain a population-based estimate for patients with lung cancer. As shown in Fig. 1, we first identified the primary diagnosis of lung cancer (total n = 96,216) using the International Classification of Diseases, 10th Version (ICD-10) codes for lung cancer(C34) among all 2016–2019 NIS samples (N = 28,484,087). Then, after patients with missing variables were excluded, we obtained sample patients (n = 92,159, National Estimates = 460,795). We collected our samples from the NIS. Although we used NIS data for the analysis, our collected samples from the NIS are entirely independent of the NIS. (Fig. 1)

### Variables

The primary outcome of this research was to investigate the characteristics of the study sample and its association with hospital charges (total dollar amount of inpatient hospital services claims to patients’ primary payer) and length of stay (unit: days). Due to the skewing of distribution for hospital charges and length of stay, we conducted a natural log of those variables. Main independent variables in this research were race (White, Black, Hispanic, Asian or Pacific Islander, Native American, Others) and the region where patients were treated (Northeast, Midwest, South, West). In addition, we adjusted various patient-level confounders. Patient characteristics included age, sex, annual median household income (0-25th, 26th to 50th, 51st to 75th, 76th to 100th percentile), primary payer (Medicare, Medicaid, Self-Pay/No Charge, Other, Private insurance), the severity of illness (No/Minor, Moderate, Major, Extreme comorbidity or complications), year of inpatient discharge and whether the patient received surgery, radiation, or chemotherapy.

### Statistical analysis

Sampling weights were used for all statistical analyses to represent nationwide patients with lung cancer. We used DISCWT variable in order to create all national estimates for all analysis used in this study. First, we examined the characteristics of the final dataset. The patient characteristics were presented as weighted frequency (percentage) or means (SD). Then we investigated the temporal trend of average hospital charges (US healthcare inflation adjusted) and length of stay for each inpatient case. All years’ hospital charges were discounted to 2016 level using Bureau of Labor Statistics’s Consumer Price Index (medical care index; y2017 1.8%, y2018 2.0%, y2019 4.6%). ANOVA tests were employed to examine the differences. Next, we investigated how race and region are associated with hospital charges and length of stay using the multivariate sample weighted linear regression analysis. Additionally, we ran the models with census division variables to figure out more specific regional variances. Census division variable is what the U.S. Census Bureau collected population information from the following regions: New England, Middle Atlantic, East North Central, West North Central, South Atlantic, East South Central, West South Central, Mountain, and Pacific. All analyses were conducted using SAS statistical software (version 9.4; SAS Institute Inc., Cary, NC, USA). All statistical tests were two-sided, and statistical significance was determined at a p-value < 0.05.

## Results

### Patient characteristics

A total of 92,159 patients with lung cancer were identified in the 2016–2019 NIS data (weighted n = 460,795, Table 1). More patients with lung cancer were found in the Southern area. More detailed characteristics of patient characteristics are presented in Table 1. The mean hospital charges and lengths of stay were $80,854 (SD = $92,147) and 6.21 days (SD = 6.14 days) (Table 1).

**Table 1:** General characteristics of the study sample

	N/Mean	%/SD
**Total Unweighted N**	92,159	
**Weighted National Estimates**	460,795	
**Race**		
White	351,975	76.4
Black	56,920	12.4
Hispanic	23,445	5.1
Asian or Pacific Islander	16,135	3.5
Native American	1,390	0.3
Others	10,930	2.4
**Region**		
Northeast	102,630	22.3
Midwest	103,700	22.5
South	185,305	40.2
West	69,160	15.0
Age*	68.7	10.5
**Sex**		
Male	227,935	49.5
Female	232,860	50.5
**Median household income**		
0-25th percentile	133,765	29.0
26th to 50th percentile	121,550	26.4
51st to 75th percentile	110,235	23.9
76th to 100th percentile	95,245	20.7
**Primary payer**		
Medicare	293,600	63.7
Medicaid	44,555	9.7
Private insurance	99,520	21.6
Self-pay	9,735	2.1
No charge	845	0.2
Other	12,540	2.7
**Severity of Illness**		
No/Minor comorbidity or complications	53,735	11.7
Moderate comorbidity or complications	156,700	34.0
Major comorbidity or complications	176,255	38.3
Extreme comorbidity or complications	74,105	16.1
**Surgery**		
Yes	108,645	23.6
No	352,150	76.4
**Chemotherapy**		
Yes	12,370	2.7
No	448,425	97.3
**Radiation**		
Yes	4,020	0.9
No	456,775	99.1
Hospital Charges [USD]*	78,010	88,588
Length of Stay [Days]*	6.21	6.14

*Continuous variable

### Temporal patterns of hospital charges and length of stay

Table 2 shows the temporal healthcare utilization trends among hospitalized patients with lung cancer between 2016 and 2019. The number of patients with lung cancer during the study periods was stable. However, hospital charges were somewhat increased, and the length of stay was decreased during the study period (p < 0.001).

**Table 2:** Temporal trend of average hospital charges & length of stay for each inpatient case

	2016	2017	2018	2019	P
Unweighted N	23,110	23,189	23,032	22,828	
Weighted N [National Estimates]	115,550	115,945	115,160	114,140	
Hospital Charges [USD]	74,453	77,129	79,535	80,932	< 0.0001
Length of Stay [Days]	6.35	6.29	6.13	6.08	< 0.0001

### Association of race and region with hospital charges and length of stay

Table 3 shows the associations of race and region with hospital charges and length of stay. We found statistically significant racial and regional disparity among hospitalized patients with lung cancer. Hispanics were charged 21.1% higher than White patients(p < 0.001), and Asians spent 12.3% more on in-hospital charges than White patients(p < 0.001). However, Native Americans spent 12.7% less on in-hospital charges than White patients(p = 0.016). Hispanics and Blacks’ higher hospital charges were likely due to increased lengths of stay (3.5%, p = 0.001, 5.6%, p < 0.001, respectively). Compared to the Northeast, Midwest and South were associated with lower hospital charges (-22.4%, -7.4%, both p < 0.001), however, the West was positively associated with higher hospital charges (24.7%, p < 0.001).

**Table 3:** Results of sample weighted linear regression: how race & region associated with healthcare utilization

	Hospital Charges		Length of Stay	
	Est.	P	Est.	P
Race				
White	Reference			
Black	0.046	< 0.0001	0.056	< 0.0001
Hispanic	0.211	< 0.0001	0.030	0.001
Asian or Pacific Islander	0.123	< 0.0001	0.038	0.001
Native American	-0.127	0.016	0.019	0.600
Others	0.107	< 0.0001	0.055	< 0.0001
**Region**				
Northeast	Reference			
Midwest	-0.224	< 0.001	-0.056	< 0.001
South	-0.074	< 0.0001	0.001	0.9192
West	0.247	< 0.0001	-0.059	< 0.0001
**Age**	-0.008	< 0.0001	0.000	0.043
**Sex**				
Female	Reference			
Male	0.029	< 0.001	0.008	0.032
**Median household income**				
76th to 100th percentile	Reference			
26th to 50th percentile	-0.057	< 0.0001	0.045	< 0.0001
51st to 75th percentile	-0.046	< 0.0001	0.024	< 0.0001
0-25th percentile	-0.066	< 0.001	0.057	< 0.001
**Primary payer**				
Private insurance	Reference			
Medicaid	0.063	< 0.0001	0.106	< 0.0001
Medicare	0.173	< 0.001	0.057	< 0.001
Self-pay	-0.007	0.730	0.062	< 0.0001
No charge	0.067	0.267	0.062	0.208
Other	-0.463	< 0.0001	-0.088	< 0.0001
**Severity of Illness**				
No/Minor comorbidity or complications	Reference			
Moderate comorbidity or complications	0.063	< 0.0001	0.259	< 0.0001
Major comorbidity or complications	0.210	< 0.0001	0.558	< 0.0001
Extreme comorbidity or complications	0.681	< 0.0001	0.919	< 0.0001
**Year**	0.064	< 0.0001	-0.002	0.367
**Surgery**				
No	Reference			
Yes	0.793	< 0.001	0.305	< 0.001
**Chemotherapy**				
No	Reference	< 0.0001	0.541	< 0.0001
Yes	0.577	< 0.001	0.541	< 0.001
**Radiation**				
No	Reference			
Yes	0.510	< 0.001	0.387	< 0.001

### Models with census division variables

Table 4 holds the results of the model where we replaced the region variable with the Census Division. In this result, we also found specific regional variances. Compared to the South Atlantic, the New England (-23.1%, < 0.0001), East North Central (-14.9%, < 0.0001), West North Central (-17.3%, < 0.0001), and East South-Central (-9.9%, < 0.0001) regions were associated with lower hospital charges. However, higher hospital charges were associated with the Middle Atlantic (18.0%, < 0.0001), West South Central (6.4%, < 0.0001), Mountain (25.3%, < 0.0001), and Pacific (34.7%, < 0.0001).

**Table 4:** Results of sample weighted linear regression: using more specific region variable (Census Division)

	Hospital Charges		Length of Stay	
	Est.	P	Est.	P
**Census Division**				
South Atlantic	Reference			
Middle Atlantic	0.180	< 0.0001	0.017	0.009
East North Central	-0.149	< 0.0001	-0.057	< 0.0001
West North Central	-0.173	< 0.0001	-0.031	0.000
New England	-0.231	< 0.001	-0.028	0.001
East South Central	-0.099	< 0.0001	-0.009	0.286
West South Central	0.064	< 0.0001	0.032	< 0.0001
Mountain	0.253	< 0.0001	-0.029	0.004
Pacific	0.347	< 0.0001	-0.064	< 0.0001

**All other variables were adjusted**

## Discussion

Racial and regional disparities for lung cancer are demonstrated here through increased hospital charges and length of stay. Our study explored various patient-related factors and found significant differences in race, insurance, and region for hospital charges and length of stay. Such findings demonstrate high need and disparity, racial differences, and socioeconomic barriers. With lung cancer carrying an increased financial burden, these findings are especially significant for resource allocation, hospital planning, and public health initiatives to improve patient outcomes.

Identifying racial health disparities may identify targeted barriers to care. For example, Hispanic patients spent the most on hospital charges despite having the shortest stays compared to Asian and Black patients. In addition, past research has shown that Hispanic patients with cancer are often treated in hospitals instead of transitional care centers and often present to the ER or ICU for aggressive chemotherapy [7, 8]. Such practices may identify an increased burden for care that Hispanic patients face compared to other races. Asian or Pacific Islanders had 12.3% higher hospital costs than White patients and had the second-highest lengths of stay. Similar to previous literature, our results signify an economic disparity for Asian patients[6]. In the Chen et al. study, Black patients were also likely to be charged more and spend longer in hospitals[6]. Our study did find that Black patients were charged more than White patients but less than Hispanic and Asian or Pacific Islander patients. However, Black patients were hospitalized longer than all other racial or ethnic groups. The expensive hospitalizations and high lung cancer mortality rate [20, 21] indicate mismatched care unique to Black patients.

Regional differences can highlight areas at increased risk for health disparities in the United States. Compared to the Midwest, all other regions had higher lengths of stay. The West had 47.1% higher hospital charges than the Midwest, making it the most expensive region for lung cancer treatment in the United States. With poverty rates in the West in 2019 between 9–11% [22] yet a median household income of $75,796 in 2019, [23] these results have grave consequences for patients. The Northeast and South had equal lengths of stay, yet care was more expensive in the Northeast. Previous research found that the New England and Middle Atlantic regions in the Northeast had some of the highest levels of neighborhoods with incomes over $63,000 [14] and the highest median income in 2019 of $76,221[23]. Further, the Northeast has wide variations in poverty rates from less than 9.5–13% [22]. For comparison, the South has some of the highest poverty rates, from 12% up to 19.6% [22].

Discussing the medical care consumer price index (CPI) may demonstrate how economic differences described in our study can impact access to care. Medical care services (professional/hospital/related services and health insurance) CPI shows how much out-of-pocket spending is conducted on these services [24] and can be compared across all four regions. In our study, the Northeast had one of the highest hospital charges, and according to the US Bureau of Labor Statistics, in December 2019, it had the highest medical services CPI at 539.822[25]. The West closely followed it at 530.506; [26] then the Midwest at 505.493; [27] and lastly the South at 479.724[28]. The high CPI in places like the Northeast and West may be manageable for the higher-income earners but could be an unreasonable burden for lower-income or impoverished patients. Though the South has the lowest medical services CPI, it still has the highest poverty rate and, in our study, had one of the most extended lengths of stay and 15% higher hospital charges than the reference group. Therefore, economic barriers may be more prevalent in the South because of increased healthcare utilization and economic differences. Economic disparities are essential to highlight because healthcare affordability can hinder care [29–31].

When we replaced the region variable with Census Division, we could make comparisons across more specific geographic regions and races. In this model, Hispanic patients were charged 18.4% more yet were hospitalized for the least amount of time than Black and Asian/Pacific Islander patients. Black patients had the most extended lengths of stay and were charged 2.7% more than white patients. Regions with the highest charges were the Middle Atlantic, West South Central, Mountain, and Pacific. Of this group, only Mountain and Pacific regions had lower lengths of stay than the reference region South Atlantic, signifying that patients in these regions are paying more for less time in the hospital. These findings are significant because regions like West South Central, especially Texas, have some of the highest diversity indexes; New York and New Jersey in the Middle Atlantic follow closely[32]. The Mountain and Pacific regions have states like California and Nevada, 2nd and 3rd in diversity indexes, and other highly diverse states like New Mexico, Arizona, and Colorado[32]. The diversity index shows the likelihood of randomly chosen people from different races[32]. When considering the diversity index and our study results, it illustrates how in places like West South Central, which has states with some of the highest diversity indexes, [32] the higher charges and lengths of stay for minorities become undue- and concentrated- burdens.

These differences in poverty rates, median incomes, and healthcare utilization by race and region may signify health equity issues. Health equity is when all patients have equal access to care, and inequalities occur when access to treatment differs [33, 34]. The income differences within each region in our study and by comparison to each other illustrate two different realities. Higher-income patients may be able to afford the extended costs seen in the Northeast, West, and South, but low-income patients may face more significant financial distress and barriers to care. Past research has demonstrated that low-income patients are less likely to have surgery for lung cancer [5] and are more likely not to be treated [12, 13]. Some minority patients may be at increased risk for such burdens because they typically have lower-income levels [35, 36]. For example, in 2019, the median income for African Americans was $41,098 for women and $45,644 for men; for Hispanic women, it was $36,110 and $41,519 for men[35]. The median household income for the United States in 2019 was $68,703[37]. Asian men in 2019 were the only minority ethnicity to earn above this income level; Black and Hispanic men and women made the least of all other races[35]. The white median household income was $72,204[23].

Health inequalities can be quantified based on differences in healthcare cost and utilization [38, 39]. Our analysis has demonstrated differences in hospital charges and length of stay between ethnicities and regions. Therefore, our study illustrates impactful health inequalities when combining increased regional utilization with the increased economic burdens and size of stay differences for minority patients with lung cancer. As a result, we have found a need to target high-risk areas and patients for financial assistance and prevention programs.

With health equity judged by access to healthcare, payment becomes an integral part of health equity as patients typically access care by paying out-of-pocket or using insurance. This study found differences in healthcare utilization by insurance. The Medicare group noted the highest prices, with charges 17.3% higher than private insurance for only 5.7% longer stays. Conversely, Medicaid had 6.3% higher costs but the most prolonged stay with patients hospitalized for 10.6% more than private insurance. Previous research shows that patients with Medicaid are more likely not to have treatment, receive nonstandard treatment, [12, 13] or forgo surgery[5]. Regarding insurance rates in 2019, there were higher rates of Medicaid coverage in Black (alone or in combo) and Hispanic (any race) populations[40]. About 29.2% of the Black population used Medicaid, 28.3% of Hispanics used Medicaid, and 13.5% of Asians used Medicaid[40]. White non-Hispanics had the lowest rates for Medicaid coverage (11.5%), with 75.2% using private insurance[40]. Consequently, although some minority patients have insurance coverage, their access, utilization, and cost of healthcare can vary widely, indicating a significant disparity in care that further fuels the health inequality faced by minorities.

Although this study has explored various aspects fueling healthcare inequality, like differences in healthcare utilization and cost by race, region, and insurance type, these findings have limitations. First, the National Inpatient Sample dataset used ICD-10 codes for lung cancer, limiting patient selection. The dataset also does not include clinical information or disease severity, restricting real-life interpretation. The race variable does not reflect multiracial information. Lastly, the annual medical services CPI could not be found for all four census regions (only for the Northeast and West), so the CPI for December 2019 was used because it was available for all four areas and was the last month of our study period. Despite these limitations, this study has found significant health disparities that warrant further research and action by policymakers or hospital managers to promote greater equality in cost and care.

## Conclusion

By comparing the length of stay and hospital charges, this study has identified health disparities by race, region that disproportionately targets minority and low socioeconomic groups. Regions identified in this study may be places of interest to target more tailored prevention programs like smoking cessation and lung cancer screenings [21, 41–43]. Specialized public health programs should be conducted to increase access to care and promote health equity amongst minority and economically disadvantaged groups. Hispanic and Black patients with lung cancer appeared to be at the most significant disadvantage because of lower median income levels, higher costs, more extraordinary lengths of stay, and a more substantial percentage of Medicaid usage. Similarly, patients in the South, especially West South Central, may face more significant disparities due to income and healthcare utilization differences. The combination of these risk factors has severe implications for patients with lung cancer and must be addressed to achieve health equity. Hospital managers and policymakers can address these concerning trends through financial assistance programs for minorities and low-income patients. Further research is needed to identify the best strategies for closing the disparity gap to increase access and the health of disadvantaged communities and at-risk regions.

## Data Availability

All data generated or analyzed during this study are included in this published article.
